# Supplementary material for: Effects of boysenberry on postprandial energy metabolism in healthy adults: A randomized controlled crossover trial
Source: PLoS One. 2025 Aug 22;20(8):e0330683. doi: 10.1371/journal.pone.0330683 (PMC12373189; doi:10.1371/journal.pone.0330683)
Supplement: S2 File — (PDF) [file pone.0330683.s006.pdf]

Research Protocol
Investigation of the Effects of Beverage Intake on Diet-Induced Thermogenesis

Planned Study Period: From the date of approval to March 31, 2024
Created: 14/01/ 2022, Ver. 1.0

(English Translation from Japanese)

Table of Contents

1. Overview
2. Background
3. Objectives
4. Overview of Test Foods
5. Subjects
6. Informed Consent
7. Research Methods
8. Observation and Examination Items
9. Outcomes
10. Termination and Dropout of the Study
11. Evaluation and Reporting of Adverse Events
12. Data Collection
13. Statistical Analysis
14. Target Sample Size and Rationale
15. Ethics
16. Quality Control and Quality Assurance
17. Research Funding and Conflict of Interest
18. Publication of Research Results and Ownership of Rights
19. Compliance with and Changes to the Research Implementation Plan
20. End and Suspension/Interruption of the Study
21. Research Implementation Structure
22. Structure and Contact Points for Consultation Regarding the Research by Subjects and Their Associates
23. References

Appendix

A. Information Document, Consent Form, Consent Withdrawal Form

Principal Investigator
Ippei Shimizu, Associate Professor, Department of Cardiology, Juntendo University

Contact Information
2-1-1 Hongo, Bunkyo-ku, Tokyo 113-8421, Japan

Co-Investigator
Ryo Furuuchi, Part-time Assistant Professor, Department of Advanced Senotherapeutics, Juntendo University

Contact Information
2-1-1 Hongo, Bunkyo-ku, Tokyo 113-8421, Japan

Collaborating Research Institutions

Laboratory for Exercise and Environment Physiology, Faculty of Education, Niigata University
Responsible Person: Tatsuro Amano
Role of Research Director: Conducting measurements and data analysis
Address: 8050 Igarashi 2-chome, Nishi-ku, Niigata 950-2181, Japan

Bourbon Corporation, Advanced Research Institute
Responsible Person: Daisuke Maejima
Role of Research Director: Preparation of test products
Address: 316-2 Higashijima, Akihabara-ku, Niigata 956-0841, Japan

2. Overview

2-1. Study Design

Multicenter, randomized, crossover, double-blind.

2-2. Objective

The objective of this study is to evaluate the effects of consuming boysenberry juice for four weeks on diet-induced thermogenesis and associated energy expenditure.

2-3. Subjects

Healthy adults.

2-4. Intervention

Based on the eligibility assessment results at the time of registration, the following interventions will be performed: Participants will consume either 100 mL of boysenberry juice or 100 mL of placebo beverage daily during the four-week study period.

2-5. Outcomes

1) Changes in postprandial thermogenesis after four weeks of intake.
2) Changes in energy expenditure after four weeks of intake.
3) Changes in body composition after four weeks of intake.
4) Changes in oxygen consumption, carbohydrate oxidation, fat oxidation, and respiratory quotient after four weeks of intake.
5) Changes in subjective indicators after four weeks of intake.

2-6. Target Sample Size and Study Period

Target sample size: 36 participants.
Study period: From the date of approval to March 31, 2024.

3. Background

Brown adipose tissue (BAT) has been identified as an important tissue for metabolic regulation throughout the body, in addition to its role as a thermogenic organ. The activity of brown adipose tissue decreases with age, and it has been reported to be significantly inversely correlated with BMI and body fat, representing a novel therapeutic target for age-related diseases such as obesity and metabolic disorders. BAT promotes energy expenditure through non-shivering thermogenesis in cold environments, and it is also suggested to contribute to energy expenditure through daily thermogenesis. Diet-induced thermogenesis (DIT), the phenomenon of heat production following food intake, is a well-known physiological process, and BAT has been reported to contribute to DIT and promote energy expenditure, indicating its potential importance in the prevention of various diseases.

Polyphenolic compounds found in foods have been reported to possess various health benefits. Some polyphenolic compounds have been shown to activate BAT, which may be utilized for therapeutic and preventive strategies targeting BAT function. Boysenberry, a berry fruit rich in polyphenols, contains anthocyanin compounds in boysenberry juice that have been found to potentially improve and protect vascular endothelial function, thereby enhancing cardiovascular function. Additionally, we have obtained data suggesting that the intake of boysenberry polyphenols may suppress or improve the decline in BAT function in mice. Additionally, preliminary investigations conducted on humans have shown that the intake of boysenberry juice for four weeks increases thermogenesis in the supraclavicular region, considered to be a site of BAT, during cold exposure, suggesting an improvement in BAT function (data not yet published, UMIN000043476). On the other hand, the effects of polyphenol intake on daily energy expenditure, such as DIT, are not well understood, and further elucidation through intervention studies involving humans is needed.

4. Objectives

Based on previous research findings, we hypothesize that the intake of boysenberry juice may increase daily energy expenditure, specifically DIT, and postprandial energy expenditure through the improvement of BAT function. This study aims to investigate the effects of the consumption of boysenberry and placebo beverages on DIT and associated postprandial energy expenditure in healthy adults through a crossover trial, thereby elucidating the impact of polyphenols.

5. Overview of Test Foods

1) Boysenberry Juice

Nutritional components per 100 mL:
Energy: 32 kcal
Carbohydrates: 7.3 g
Protein: 0.4 g
Fat: 0.1 g
Sodium: 0 mg
Total Polyphenols: 294 mg
Anthocyanins: 79 mg

2) Placebo Beverage

The placebo beverage was designed to match the carbohydrate and organic acid content of the boysenberry juice in 1), and the appearance and flavor were adjusted using coloring agents and flavoring agents to be similar to that of 1).

Safety Information

Boysenberry juice is a commercially available beverage with no reported adverse events to date, indicating that there are no safety concerns.

6. Subjects

6-1. Study Participants

Healthy adult males and females.

6-2. Recruitment Method

Participants will be broadly recruited through the posting of posters and distribution of leaflets at various locations such as companies, universities, sports clubs, and health clubs, allowing for voluntary participation.

6-3. Inclusion Criteria

1. Males and females aged 20 years or older at the time of consent.
2. Individuals who are healthy and do not have any chronic diseases.
3. Individuals with a BMI of 18.5 or higher and less than 25.0.
4. Individuals who have provided written informed consent to participate in the study.

Rationale for Setting Criteria

1. To target capable and healthy adults for informed consent.
2. To ensure evaluation in a population similar to that encountered in routine clinical practice, no upper age limit is set.
3. The criteria have been established in accordance with ethical guidelines.

6-4. Exclusion Criteria

1. Individuals with any diseases who are undergoing drug treatment.
2. Individuals suffering from severe diseases or allergic conditions.
3. Individuals who regularly consume supplements or health foods during the study period.
4. Individuals who excessively consume foods containing polyphenols during the study period.
5. Individuals who are expected to undergo significant changes in exercise habits, job, or academic lifestyle during the study period.
6. Pregnant or nursing women.
7. Individuals deemed unsuitable by the principal investigator.

Rationale for Setting Criteria

1. To target healthy adults.
2. Because the effective component of the test food is considered to be polyphenols.
3. Because lifestyle factors such as exercise habits and diet may influence the study outcomes.

7. Informed Consent

In conducting this study, informed consent will be obtained in accordance with the "Ethical Guidelines for Life Science and Medical Research Involving Human Subjects."

7-1. Procedures for Obtaining Informed Consent

A consent explanation document approved by the Ethics Committee for Research Involving Human Subjects at the Medical School will be provided to the research participants (including their legal representatives, if necessary). Adequate explanations will be given both in writing and orally, and written informed consent will be obtained based on the participants' free will. If any information arises that may affect the

participants' consent or if there are changes to the research protocol that may influence the participants' consent, the participants will be promptly informed, and their willingness to continue participation in the study will be confirmed beforehand. Any revisions to the consent explanation document will also be made with prior approval from the Ethics Committee, and re-consent from the participants will be obtained.

The consent explanation document will include the following information:

1. The title of the research and a statement confirming that permission has been obtained from the head of the research institution to conduct the study.
2. The name of the research institution and the name of the principal investigator (including the names of the collaborating research institutions and their principal investigators if the research is conducted in collaboration with other institutions).
3. Purpose and Significance of the Research
4. Research Methods (including the purpose of utilizing samples and information obtained from research participants) and Duration
5. Rationale for Selecting Research Participants
6. Burden on Participants and Anticipated Risks and Benefits
7. Statement that Consent to Participate or Continue in the Research Can Be Withdrawn at Any Time (including information on the circumstances and reasons if such withdrawal would make it difficult to implement measures based on the withdrawal)
8. Assurance that Participants Will Not Be Treated Unfavorably for Refusing or Withdrawing Consent to Participate or Continue in the Research
9. Method of Information Disclosure Regarding the Research
10. Statement that Research Plan and Methodology Materials Can Be Obtained or Accessed at the Request of Research Participants, Provided that It Does Not Hinder the Protection of Personal Information of Other Research Participants and the Preservation of the Study's Originality, along with Information on How to Obtain or Access This Material
11. Handling of Personal Information (including methods of anonymization, and a statement regarding the creation of anonymized or non-identifiable information)
12. Methods for the Storage and Disposal of Samples and Information
13. Source of Funding and Status of Conflicts of Interest Related to the Research Institution and Individual Researchers
14. Handling of Results Obtained from the Research
15. System and Contact Points for Research Participants and Their Associates to Consult

Regarding the Research (including genetic counseling)

16. Disclosure of Any Economic Burden or Compensation to Research Participants, Including the Details and Nature of Such Compensation

7-2. If Information is Obtained that May Affect Participation in the Research

If the research physician obtains any information during the study that may influence a participant's decision to continue their involvement in the study, they will immediately explain that information to the participant and confirm their willingness to continue participation. At the same time, the consent explanation document will be revised, and approval will be sought from the Ethics Committee to obtain written consent from the participant for continued participation in the study.

8. Research Methods

8-1. Study Design

Multicenter, randomized, crossover, double-blind.

8-2. Outline of the Study

The researcher will obtain consent from the participants and confirm their eligibility before commencing the trial. The duration of participants' involvement in the study will be as follows:

1. Eligibility assessment and consent form acquisition.
2. Consumption of the test food for four weeks, with testing conducted in the fourth week of consumption.
3. Washout period of four weeks.
4. Change of test food, followed by the consumption of the new test food for four weeks, with testing conducted in the fourth week of consumption.
5. Study conclusion.

A schematic outline is provided below.

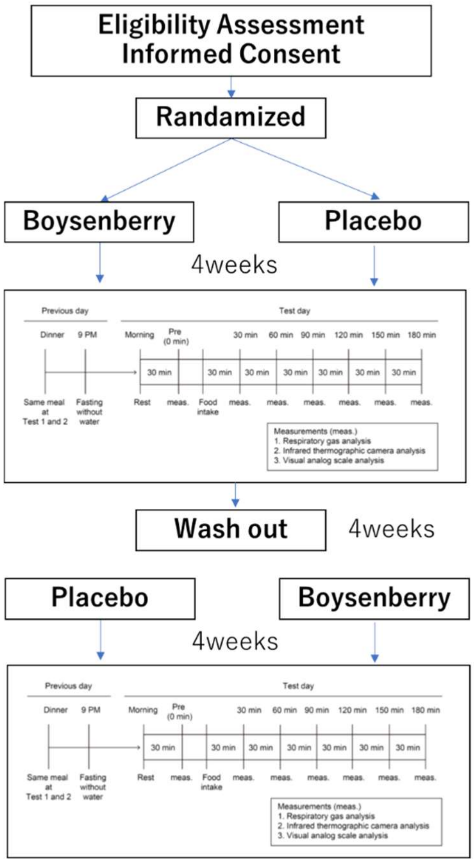

8-3. Target Sample Size and Study Period

Target sample size: 36 participants (6 participants from Juntendo University, 30 participants from Niigata University)

Maximum enrollment period: From the date of approval to March 31, 2024
Study period: From the date of approval to March 31, 2024

8-4. Intervention Methods

During the consumption period of the test food, participants will be instructed to consume 100 mL of the test food at their discretion throughout the day. Each test food will be consumed for four weeks. Participants will be required to keep a record of their intake during the study period. Participants are advised to refrain from strenuous exercise the day before testing and to consume a specified meal by the study conductors by 21:00. After 21:00, participants should arrive at the testing site in a fasting state, except for water, to undergo the testing.

8-5. Expected Duration of Participation for Research Subjects

Research participants will participate for a period of 12 weeks after providing consent.

9. Examination Items

9-1. Examination Schedule

Examination Items	Consent Acquisition	Eligibility Confirmation	1st Test Day	2nd Test Day	Test Food Consumption Period
Informed Consent	□				
Background of Participants	□				
Consumption of Specified Dinner			□		
Restriction of Strenuous Exercise			□		
Fasting After 21:00			□		
Height and Weight	□		□	□	□
Dietary Intake				□	□
Thermography				□	□
Respiratory Gas Analysis				□	□
Visual Analog Scale (VAS)				□	□
Dietary Record					□

9-2. Examination Items

1) Background of Participants

- The following items will be investigated:
- Gender, date of birth, smoking and drinking status
- Presence of chronic diseases
- Use of medication
- Use of supplements
- Intake of polyphenols
- Presence of allergic conditions
- Pregnancy status
- Height and weight
- Changes in lifestyle habits during the study period (such as changes in exercise habits and workplace)

2) Measurement of Height and Weight

Participants will have their height and weight measured after arriving at the testing site.

3) Resting Measurements (Respiratory Gas Analysis and Thermography Camera

Imaging)
Measurements will be conducted in a laboratory set at 27 ± 1°C. Participants will wear light clothing consisting of a tank top with an open neckline and shorts, and will remain at rest for 30 minutes. After resting, baseline skin temperatures of the supraclavicular area and fingertips will be recorded using a thermography camera. Following this, respiratory gas analysis will be performed.

4) Dietary Intake
After the resting measurements, participants will consume a specified meal. The meal will be provided to account for 15% of the total energy expenditure based on the Harris-Benedict Calculator, and participants will consume it with 200 mL of water.

5) Postprandial Measurements
Measurements will be taken 30, 60, 90, 120, 150, and 180 minutes after the start of the meal, including thermography camera imaging of the supraclavicular area and fingertips, as well as respiratory gas analysis.

6) Visual Analog Scale (VAS) Assessment
Participants will be handed a VAS assessment form before the meal starts and again after the 180-minute postprandial measurement to record their subjective indicators.

7) Dietary Record
After the trial begins, participants will be required to maintain a dietary record diary.

9-3. Random Allocation
An allocation officer will be designated separately from the trial personnel (study director, trial implementers, and data analysts) to manage the order of treatment allocation for the participants. Participants will be allocated based on stratified randomization considering factors that may influence brown adipose tissue function, such as age, BMI, and gender. Allocation will be performed using a random number table, and the allocation information will not be disclosed to the trial implementers and participants during the study (ensuring double blinding). The allocation information will be revealed to the trial implementers by the allocation officer after all measurements for the participants have been completed and after a predetermined period.

10. Outcomes

10-1. Primary outcome

Changes in postprandial thermogenesis and energy expenditure after four weeks of intake.

10-2. Secondary outcomes

2) Changes in body composition after four weeks of intake.
3) Changes in oxygen consumption, carbohydrate oxidation, fat oxidation, and respiratory quotient after four weeks of intake.
4) Changes in subjective indicators after four weeks of intake.

11. Termination and Dropout from the Study

11-1. Criteria for Study Termination

The study will be terminated if any of the following events occur after registration. When terminating the study, observations, tests, and evaluations will be conducted as promptly as possible.

1) If an adverse event occurs that leads the principal investigator to determine that continuing the study is not feasible.
2) If a participant requests to withdraw from the study.
3) If, for any other reason, the principal investigator determines that continuing the study is not feasible.

11-2. Study Dropout

Participants will withdraw from the study and no further investigations will be conducted if any of the following occur after registration:

1) The participant cannot continue the study due to personal circumstances (such as relocation) or other reasons.
2) The participant requests to terminate their participation in the study or withdraws consent.In such cases, the participant's intent to withdraw from the study will be obtained using the "Consent Withdrawal Form" (Appendix).

12. Response to Adverse Events

12-1. Adverse Events

An adverse event refers to any undesirable or unintended health problem or symptom experienced by a participant, regardless of its causal relationship with the research. In the event of an adverse event, the responsible physician will take prompt and appropriate action to ensure the participant's safety. Additionally, if treatment for the adverse event is necessary, the participant will be informed accordingly.

12-2. Reporting of Adverse Events
In the event of a serious adverse event, the responsible physician will promptly report it in writing to the head of the research institution. Additionally, communication for reporting in accordance with the safety information reporting system for pharmaceuticals and medical devices, as stipulated in the "Law on Securing Quality, Efficacy, and Safety of Pharmaceuticals and Medical Devices," will be carried out appropriately in accordance with the regulations of each research facility.

13. Data Collection

13-1. The investigator will appropriately record and store the investigation items.

13-2. Data Management
Data modification and database management will be conducted, with the creation of statistical analysis datasets performed at Juntendo University.

14. Statistical Analysis

Measurements and changes will be compared using appropriate statistical analysis methods such as paired t-tests, analysis of variance (ANOVA), and multiple comparison tests.

15. Target Sample Size and Rationale

36 participants.

[Rationale for Setting Criteria]
In a preliminary open-label, before-and-after comparison study involving 10 participants, the effects of the test food on brown adipose tissue were evaluated. The increase in skin temperature in the supraclavicular area during cold exposure before and after the consumption of the test food was used as an indicator to analyze the function of brown adipose tissue. Based on this data, the sample size was calculated with a power of 95%

and a significance level of 0.05, resulting in a calculated sample size of 22 participants. However, since this study uses different indicators than the preliminary trial, previous literature was referenced, and significant differences were observed in a crossover trial involving 36 participants. Therefore, it was assumed that a sample size of more than 22 and less than 36 participants would be necessary to obtain significant differences in this study, leading to the establishment of the sample size of 36.

16. Ethics

This study will be conducted in accordance with the principles of the latest Declaration of Helsinki to protect the rights and welfare of patients, and in compliance with the guidelines of the "Ethical Guidelines for Medical Research Involving Human Subjects" issued by the Ministry of Education, Culture, Sports, Science and Technology and the Ministry of Health, Labour and Welfare.

16-1. Ethical Review

The responsible physician will seek the opinions of the ethics review committee of the research representative's affiliated institution and obtain permission from the head of the research institution prior to conducting the study.

16-2. Protection of Personal Information

Individuals involved in the conduct of this study will take appropriate measures to ensure the privacy and protection of participants' personal information. The registered participant information will be anonymized by assigning a registration number specifically for this study. Reports will not include any identifiable information such as names or numbers. When publishing the results of the study, no information that could identify participants will be included. Data obtained from participants during the study will not be used for purposes other than those of this research; if there is a need to use it for purposes other than research, separate consent will be obtained from the participants as necessary.

16-3. Compensation for Health Damages

If any health damages occur to participants as a result of this study, the research facility will take necessary measures, including treatment. The costs of treatment will be covered by the health insurance of the participants.

16-4. Benefits and Risks for Participants

The results of this study may establish a method to improve the efficiency of postprandial

thermogenesis and energy expenditure through the intake of food components, thus improving the balance of energy intake and expenditure. Improving this balance is important for the prevention of various diseases associated with obesity and aging, and it is expected to contribute to the advancement of medicine in the future. The test foods used in this study are within the range of what is typically consumed in a normal diet, and it is believed that the risk of experiencing any disadvantages is extremely low.

16-5. Financial Burden on Participants

The costs associated with the trials conducted on research participants will be funded by the collaborative research funds from Juntendo University, Niigata University, and Bourbon Corporation. Participants who cooperate through to the end will receive a compensation fee (7,000 yen per day for measurements over 2 days plus a burden fee of 6,000 yen for food intake, totaling a 20,000 yen voucher). Payments will be appropriately managed through receipts and payment records.

17. Quality Control and Quality Assurance

17-1. Data Handling

Trial data will be stored at Juntendo University for ten years from the date of the report on the discontinuation or completion of the study.

17-2. Record Keeping

Documents related to the study, such as consent forms, participant number correspondence tables, examination data, and approval letters from the ethics review committee, will be stored at the research implementing facility and will be discarded ten years after the study is discontinued or completed.

17-3. Monitoring and Auditing

Regarding Monitoring Implementation

Self-monitoring will be conducted at the start, during any changes, at the end, and at least once a year.

Self-Monitor: Ryo Furuuchi

Regarding Auditing Implementation

No auditing will be conducted. Monitoring will be carried out in accordance with the

"Procedures for the Implementation of Monitoring and Auditing in Medical Research Involving Human Subjects" (Clinical Research Support Center).

17-4. Reporting to the Head of the Research Institution

Researchers will promptly report to the head of the research institution if significant concerns arise regarding the leakage of information related to the research, ethical considerations for the rights of research participants, or issues concerning the conduct of the research.
Researchers will promptly report to the head of the research institution if they obtain information that may compromise the appropriateness of the research implementation or the trustworthiness of the research results.
The principal investigator will report to the head of the research institution without delay if they obtain any facts or information that may undermine the ethical validity or scientific rationality of the research, which may impact the continuation of the study (except in cases referred to in point 4).
The principal investigator will report the progress of the research and the occurrence of adverse events associated with the study to the head of the research institution as stipulated in the research protocol.
The principal investigator will report necessary matters to the head of the research institution when the research is concluded (including in cases of termination).

18. Research Funding and Conflict of Interest

18.1. Research Funding

This study will be conducted using the collaborative research funds from Juntendo University, Niigata University, and Bourbon Corporation, with funding provided by Bourbon Corporation. Additionally, individuals affiliated with the collaborative research course at Juntendo University, funded by Bourbon, are involved in this study.

18.2. Conflict of Interest

The principal investigator and co-investigators include members affiliated with Bourbon Corporation, the funding provider, as well as individuals in the collaborative research course at Juntendo University funded by Bourbon. However, measurements will be carried out by investigators from Niigata University who do not have a direct conflict of interest with Bourbon to ensure that Bourbon does not have any involvement in these

aspects. Furthermore, blinding will be maintained until all data analyses are completed to prevent any potential bias that could favor Bourbon Corporation. All researchers will manage conflicts of interest appropriately in accordance with their respective organizations' conflict of interest management policies and will disclose such conflicts upon request when presenting research results at conferences or publishing in medical journals.

19. Publication of Research Results and Ownership of Rights

19-1. Clinical Trial Registration

This study will be registered with the UMIN Clinical Trials Registry (http://www.umin.ac.jp/ctr/index-j.htm) for public information. The clinical trial registration will be conducted by the research institution or the research office before the enrollment of the first participant.

19-2. Publication of Research Results and Attribution of Findings

The principal investigator will publish the results of this study promptly after its completion. Those presenting the research findings must obtain prior review and approval from the principal investigator, the lead researcher, and the research team. Authors of papers and conference presentations will be determined appropriately by the principal investigator and lead researcher in accordance with the authorship criteria of the International Committee of Medical Journal Editors. All data obtained from this study will belong to the research organization.

20. Compliance with and Changes to the Research Implementation Plan

20-1. Deviations from the Research Implementation Plan

The research physician may not deviate from or make changes to the research implementation plan without prior approval from the head of the research institution, based on the approval from the the Ethics Review Committee at Juntendo University, except in cases where it is necessary to avoid immediate danger to the participants.

21. Termination and Suspension of the Study

21-1. Study Termination

At the conclusion of the study, the research physician will report in writing to the head of

the research institution.

21-2. Termination and Suspension of the Study

If the study is terminated or suspended due to the decision or circumstances of the research implementing facility, the research physician will promptly report this to the principal investigator.

Response in the Event of Study Termination

The principal investigator or co-investigators will terminate the study for individual participants if they determine that continuation of the study for those participants is not feasible for the following reasons. In such cases, the reason for termination will be explained to the participants as necessary. Furthermore, the treatment for participants after termination will be managed diligently to ensure that it does not lead to any disadvantage for them.

Criteria for Termination

1) If the participant requests to withdraw from the study or withdraws consent.
2) If pregnancy is confirmed.
3) If the entire study is terminated.
4) If, for any other reason, the investigator determines that termination of the study is appropriate.

22. Support System and Contact Points for Consultation by Research Participants and Their Associates

Consultations from research participants and their associates will be addressed through the following contact points:

Contact Points

Principal Investigator

Ippei Shimizu, Associate Professor, Department of Cardiology, Juntendo University

2-1-1 Hongo, Bunkyo-ku, Tokyo 113-8421, Japan

Niigata University, Faculty of Humanities and Social Sciences

Tatsuro Amano

8050 Igarashi 2-chome, Nishi-ku, Niigata 950-2181, Japan

Bourbon Corporation
Daisuke Maejima
8050 Igarashi 2-chome, Nishi-ku, Niigata 950-2181, Japan

23. References

N Engl J Med 2009; 360:1500-1508
Int J Obes (Lond). 2021 Nov;45(11):2499-2505.
J Nutr Biochem. 2019 Feb;64:1-12
British Journal of Nutrition (2010), 103, 775–780
