# Supplementary material for: Effects of boysenberry on postprandial energy metabolism in healthy adults: A randomized controlled crossover trial
Source: PLoS One. 2025 Aug 22;20(8):e0330683. doi: 10.1371/journal.pone.0330683 (PMC12373189; doi:10.1371/journal.pone.0330683)
Supplement: S3 File — (PDF) [file pone.0330683.s007.pdf]

飲料摂取が食事誘発性熱産生に
与える影響の検証

研究計画書

研究実施予定期間:研究実施許可日～2024/3/31

作成:2022 年　1 月　14 日　Ver.1.0

目次

1. 研究実施体制
2. 概要
3. 背景
4. 目的
5. 試験食の概要
6. 対象
7. インフォームド・コンセント
8. 研究方法
9. 観察および検査項目
10. 評価項目
11. 研究の中止・脱落
12. 有害事象の評価と報告
13. データ収集
14. 統計解析
15. 目標例数と設定根拠
16. 倫理
17. 品質管理・品質保証
18. 研究資金および利益相反
19. 研究成果の公表および権利の帰属
20. 研究実施計画書の遵守と変更
21. 研究の終了および中止・中断
22. 研究実施体制
23. 研究対象者等及びその関係者が研究に係る相談を行うことができる体制及び相談窓口
24. 参考文献

付録

A. 説明文書、同意文書、同意撤回書

１．研究実施体制

本研究は以下に記載の体制で実施する。

【研究代表者】

順天堂大学 循環器内科学講座　准教授　清水逸平

【連絡先】

〒113-8421　東京都文京区本郷 2-1-1

【本学における実施体制】

【研究責任者】

順天堂大学 循環器内科学講座　准教授　清水逸平

【研究分担者】

順天堂大学　先進老化制御学講座　非常勤助教　古内　亮

【連絡先】

〒113-8421　東京都文京区本郷 2-1-1

【共同研究機関】

⑴新潟大学　新潟大学人文社会科学系（教育学部）

責任者名：天野達郎

研究責任者の役割：測定の実施・データ解析

住所：〒950-2181　新潟県新潟市西区五十嵐 2 の町 8050 番地

⑵株式会社ブルボン　先端研究所

責任者名：前島大輔

研究責任者の役割：試験品の準備

住所：〒956-0841　新潟県新潟市秋葉区東島 316 番地 2

2 概要

2-1 研究デザイン

多施設、ランダム化、クロスオーバー、2 重盲検

2-2 目的

ボイセンベリージュース摂取 4 週間が食事誘発性熱産生およびそれに伴うエネルギー消費に与える影響を評価することを目的とする。

2-3 対象

健常成人

2-4 介入

登録時の適格性判断結果に従い以下の介入を行う。試験食ボイセンベリージュース 100mL もしくはプラセボ飲料 100mL をそれぞれ 4 週間の試験期間中、毎日摂取していただく。

2-5 評価項目

１） 4 週間摂取後の食後熱産生変化

２） 4 週間摂取後のエネルギー消費量変化

３） 4 週間摂取後の体組成変化

４） 4 週間摂取後の酸素消費量、糖質酸化量、脂質酸化量および呼吸商変化

５） 4 週間摂取後の体感指標変化

2-6 目標例数と研究実施期間

１） 目標例数：36 件

２） 研究実施期間： 研究実施許可日～2024 年 3 月 31 日

3 背景

褐色脂肪組織は熱産生器官としての役割に加え、全身の代謝制御に重要な組織であることがわかっている[1)]。褐色脂肪組織の活性は加齢に伴い低下し BMI や体脂肪と有意に逆相関することが報告されており、肥満や代謝異常などの加齢性疾患の新たな治療標的と認識されている。褐色脂肪は寒冷環境下において非ふるえ熱産生を介してエネルギー消費を促すが、寒冷環境下だけではなく日常的な熱産生を介したエネルギー消費にも寄与していることが示唆されている。食事摂取後に熱を産生する食事誘発性熱産生（DIT）はよく知られた生理現象であるが、褐色脂肪は DIT に寄与しエネルギー消費を促すことが報告されており[2)]、様々な疾患の予防に重要であることが示唆される。

食品に含まれるポリフェノール成分は様々な健康効果を有していることが報告されている。いくつかのポリフェノール成分は褐色脂肪を活性化することが報告されており褐色脂肪機能を標的とした治療・予防に利用できるかもしれない[3)]。ボイセンベリーはポリフェノールを豊富に含むベリー系果実であり、ボイセンベリージュースに含まれるアントシアニン成分は、血管内皮機能を改善・保護することで循環器機能を改善する可能性を明らかにしている。また、我々はボイセンベリーポリフェノールの摂取がマウスの褐色脂肪の機能低下を抑制・改善することを示唆するデータを得ている。また、ヒトに対する予備的な検証を実施し、ボイセンベリージュースの 4 週間の摂取が寒冷負荷時にヒトの褐色脂肪領域と考えられる鎖骨上部の熱産生を上昇させることが示され、褐色脂肪の機能を改善することが示唆されている（データ未発表、UMIN000043476）。一方でポリフェノールの摂取が DIT のような日常的なエネルギー消費に与える影響はよくは分かっておらず、ヒトを対象とした介入試験による解明が求められている。

4 目的

我々はこれまでの研究結果からボイセンベリージュースの摂取は、褐色脂肪の機能改善を介して日常的なエネルギー消費である DIT および食後のエネルギー消費を上昇させるのではないかと考えた。本研究では健常成人に対しボイセンベリー及びプラセボ飲料摂取の介入を行い DIT および DIT に伴う食後のエネルギー消費への影響をクロスオーバー試験により比較し、ポリフェノールの影響を明らかにすることを目的とする。

5 試験食の概要
1) ボイセンベリージュース
栄養成分　100ml 当り
エネルギー： 32 kcal、炭水化物：7.3ｇ、タンパク質：0.4g、脂質：0.1ｇ、ナトリウム：0mg、総ポリフェノール：294mg、アントシアニジン：79mg
2) プラセボ飲料
糖質・有機酸量を 1)の試験食と揃え、着色料・香料を用い外観・味覚を１）と同様に調整した飲料。

［安全性に関して］
　ボイセンベリージュースは市販されている飲料用ジュースであり、これまでに有害事象の報告はないことから、安全性に問題はない。

6 対象
6-1 対象被験者
健常成人男女

6-2 募集方法
企業、大学、スポーツクラブや健康クラブ等の様々な場所にてポスターの掲示やリーフレットの配布を行い、自由意思により参加いただける被験者を広く公募するする。

6-3 選択基準
1) 同意取得時の年齢が 20 歳以上の男女
2) 健康な者で、慢性的な疾患がない者
3) BMI が 18.5 以上、25.0 未満の方
4) 研究参加に対して本人から文書による同意が得られた方
【設定根拠】
１）　同意取得が可能な健常な成人を対象とするため。
２）　日常診療と同様の対象集団で評価するために年齢の上限は設定しない。
３）　倫理指針に準拠して設定した。

6-4 除外基準

1） 何らかの疾患を患い薬物による治療を行っている方
2） 重篤な疾患、アレルギー疾患に罹患している方
3） 試験期間中、サプリメントなどの健康食品を常用する方
4） 試験期間中、ポリフェノールを含んだ食品を過剰に摂取する方
5） 試験期間中、運動習慣や仕事・学業など生活習慣が大きく変化する予定がある方
6） 妊産婦
7） 試験責任医師が不適当と判断した方

【設定根拠】

1） 健常成人を対象としているため
2） 本試験食の有効成分がポリフェノール成分と考えられるため
3） 運動習慣・食事などの生活習慣がアウトカムに影響する可能性があるため

7 インフォームド・コンセント

本研究を実施するときは、「人を対象とする生命科学・医学系研究に関する倫理指針」に基づいてあらかじめインフォームド・コンセントを受ける。

7-1 インフォームド・コンセントを受ける手続き

医学部医学系研究等倫理委員会で承認の得られた同意説明文書を研究対象者（代諾者が必要な場合は代諾者を含む、以下同じ）に渡し、文書および口頭による十分な説明を行い、研究対象者の自由意思による同意を文書で取得する。研究対象者の同意に影響を及ぼす情報が得られたときや、研究対象者の同意に影響を及ぼすような研究計画書等の変更が行われるときは、速やかに研究対象者に情報提供し、研究に参加するか否かについて研究対象者の意思を予め確認するとともに、事前に医学部医学系研究等倫理委員会の承認を得て同意説明文書等の改訂を行い、研究対象者の再同意を得ることとする。

同意説明文書には、以下の内容を含むものとする。

①研究の名称及び当該研究の実施について研究機関の長の許可を受けている旨
②研究機関の名称及び研究責任者の氏名（他の研究機関と共同して研究を実施する場合には、共同研究機関の名称及び共同研究機関の研究責任者の氏名を含

む。）
③研究の目的及び意義
④研究の方法（研究対象者から取得された試料・情報の利用目的を含む。）及び期間
⑤研究対象者として選定された理由
⑥研究対象者に生じる負担並びに予測されるリスク及び利益
⑦研究が実施又は継続されることに同意した場合であっても随時これを撤回できる旨（研究対象者等からの撤回の内容に従った措置を講じることが困難となる場合があるときは、その旨及びその理由）
⑧研究が実施又は継続されることに同意しないこと又は同意を撤回することによって研究対象者等が不利益な扱いを受けない旨
⑨研究に関する情報公開の方法
⑩研究対象者等の求めに応じて、他の研究対象者等の個人情報等の保護及び当該研究の独創性の確保に支障がない範囲内で研究計画書及び研究の方法に関する資料を入手又は閲覧できる旨並びにその入手又は閲覧の方法
⑪個人情報等の取扱い（匿名化する場合にはその方法、匿名加工情報又は非識別加工情報を作成する場合にはその旨を含む。）
⑫試料・情報の保管及び廃棄の方法
⑬研究の資金源等、研究機関の研究に係る利益相反及び個人の収益等、研究者等の研究に係る利益相反に関する状況
⑭研究により得られた結果等の取扱い
⑮研究対象者等及びその関係者が研究に係る相談を行うことができる体制及び相談窓口（遺伝カウンセリングも含む）
⑯研究対象者等に経済的負担又は謝礼がある場合には、その旨及びその内容

7-2 研究への参加に影響する可能性のある情報が得られた場合

研究担当医師は、研究実施中に本研究の継続参加について被験者の意思に影響を与える可能性のある情報を入手した場合には、直ちに当該情報を被験者に説明し、あらためて研究への参加継続の意思を確認する。同時に説明文書の改訂を行い倫理審査委員会の承認を得て、被験者より研究への参加継続の同意を文書で取得する。

8 研究方法

8-1 研究デザイン

多施設、ランダム化、クロスオーバー、2 重盲検

8-2 研究のアウトライン

研究担当医師は、対象患者から同意を取得し適格性を確認した後、試験を開始する。被験者の研究参加期間は、以下の期間とする。

① 適格性診断および同意書の取得
② 試験食を 4 週間摂取、摂取 4 週目に試験
③ ウォッシュアウト期間 4 週間
④ 試験食を変更し 4 週間摂取、摂取 4 週目に試験
⑤ 終了

概略図を以下に示す。

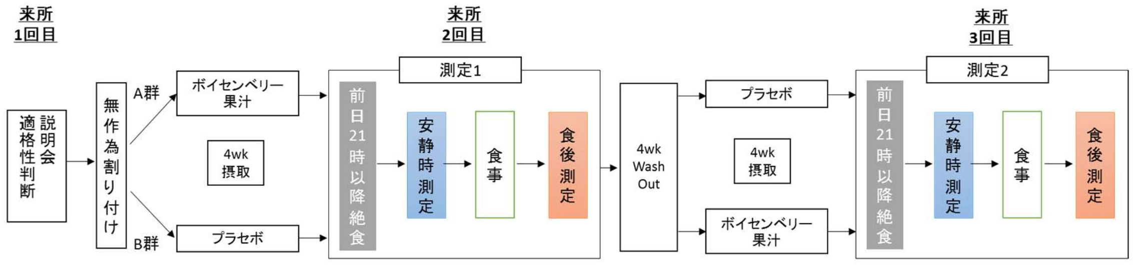

8-3 目標例数と研究実施期間

目標症例数：36 例（順天堂大学 6 例、新潟大学 30 例）

・最大登録可能期間:研究実施許可日~2024 年 3 月 31 日

・研究実施期間:研究実施許可日~2024 年 3 月 31 日

8-4 介入方法

試験食摂取期間中、被験者には 1 日のうち自由な時間に 100mL の試験食を摂取していただく。試験食それぞれを 4 週間ずつ摂取していただく。試験期間中は摂取記録をつけていただく。試験日前日には激しい運動は控えていただき、夕食は試験実施者が指定した食事を 21 時までに摂取いただく。21 時以降は水以外の飲食を控えた状態で試験会場へ来ていただき試験を受けていただく。

8-5 研究対象者の研究参加予定期間

研究対象者は同意後、12 週間の期間で参加する

9 検査項目

9-1 検査スケジュール

	同意取得<br>適格性確認	試験日前日	1 回目試験	2 日目試験	試験食摂取期間
同意取得	○				
被験者背景	○				
指定夕食の摂取		○			
激しい運動の制限		○			
21 時以降の絶食		○			
身長・体重	○		○	○	
食事摂取			○	○	
サーモカメラ撮影			○	○	
呼気ガス分析			○	○	
VAS			○	○	
食事記録					○

9-2 検査項目

1） 被験者背景

以下の項目を調査する。

- 性別、生年月日、喫煙・飲酒状況
- 慢性的疾患の有無
- 薬物治療の有無
- サプリメント摂取の有無
- ポリフェノール摂取量の調査
- アレルギー疾患の有無
- 妊娠の有無
- 身長・体重

・ 試験期間中の生活習慣の変化の有無（運動習慣や職場等の変化）

２）身長体重測定

被験者は試験会場へ来た後に身長・体重測定を実施する。

３）安静時測定（呼気ガス分析・サーモグラフィーカメラ撮影）

試験会場は 27±1℃に設定した実験室にて実施する。被験者は用意した首元の空いたタンクトップと短パンズボンの軽装になっていただき 30 分安静にする。安静の後、ベースラインの鎖骨上部および手指の皮膚温度をサーモグラフィーカメラにより撮影する。その後、呼気ガス分析を実施する。

４）食事摂取

安静時測定後、被験者は既定の食事を摂取する。食事は Harris-Benedict Calculator をもとに総エネルギー消費量の 15%のカロリーとなるように食事を提供し、200ml の水と一緒に摂取していただく。

５）食後測定

食事開始後 30, 60, 90, 120, 150, 180 分後に鎖骨上部および手指の皮膚温度のサーモグラフィーカメラ撮影と呼気ガス分析を実施する。

６）VAS 試験

食事開始前と食後 180 分測定後に VAS 試験の用紙を渡し体感指標に関して記入していただく。

７）食事記録

試験開始後、被験者には食事記録日誌を記録していただく。

9-3　無作為割り付け

本試験実施関係者（試験責任者、試験実施者、データ解析者）とは別に割付師を設定し試験食の摂取の順番の割付を依頼する。被験者は褐色脂肪機能へ影響が考えられる年齢、BMI、性別をもとに層別無作為化により割付けられる。割付は乱数表をもとに実施し、試験実施者および被験者には試験食の割付情報は試験実施中、開示されないようにする（2 重盲検化）。割付情報は被験者全ての測定が終了し一定期間の後に割付師より試験実施者へ開示される。

10 評価項目

10-1 主要評価項目

1) 4 週間摂取後の食後熱産生およびエネルギー消費量変化

10-2　副次評価項目
１）　4 週間摂取後の体組成変化
２）　4 週間摂取後の酸素消費量、糖質酸化量、脂質酸化量および呼吸商変化
３）　4 週間摂取後の体感指標変化

11 研究の中止・脱落
11-1 研究の中止基準
　登録以降に以下のいずれかの事項が発生した場合は、研究を中止する。研究中止時には、可能な限り速やかに観察、検査、評価を行う。
1)　有害事象の発現により、担当医師が研究の継続が困難と判断した場合
2)　被験者から研究を辞退する申し出があった場合
3)　上記以外の理由により、担当医師が研究の継続が困難と判断した場合

11-2 研究の脱落
登録以降に以下のいずれかの事項に該当する場合は研究から離脱し、以降の調査は行わない。
1)　被験者の都合(転居など)もしくはその他の理由により研究が継続できない場合
2)　被験者から研究参加の中止または同意撤回の申し出があった場合
「同意撤回書」(付録)を用いて被験者本人より研究離脱の意思を取得する。

12 有害事象の発生時の対応
12-1 有害事象について
　有害事象とは、被験者に生じたすべての好ましくないまたは意図しない傷病および徴候のことであり、研究との因果関係の有無を問わない。有害事象が発現した場合、担当医師は被験者に対して速やかに適切な処置を行って被験者の安全の確保に努める。また、有害事象に対する治療が必要となった場合には、被験者にその旨を伝える。

12-2 有害事象の報告
　重篤な有害事象の発現を認めた場合、担当医師は文書により遅滞なく研究機関の長に報告する。また、「医薬品、医療機器等の品質、有効性及び安全性の確

保等に関する法律」に基づく医薬・医療機器等安全性情報報告制度による報告、および製造販売業者の自発報告に協力するための連絡は、各研究実施施設の規定に沿って適切に行う。

13 データ収集

13- 1 担当医師は、調査項目を、適切に記録し保存する。

13-2 データマネジメント

データ修正、データベース管理を行い、統計解析用データセット作成は順天堂大学で行われる。

14 統計解析

測定値、変化量を対応のある t 検定、分散分析及び多重比較検定を用い比較する。

15 目標症例数と設定根拠

36 例

[設定根拠]

10 名の予備的なオープンラベル前後比較試験の検証試験において本試験食摂取の褐色脂肪への影響を評価した。試験食摂取前後の寒冷負荷時の鎖骨上部の皮膚温度上昇を指標とし褐色脂肪の機能を解析したデータを元にサンプルサイズを計算した。検出力 95%、有意水準 0.05 に設定し計算した結果、サンプルサイズは 22 名と計算された。一方で、本研究では予備的な試験とは異なる指標を用いるため先行文献[4)]を参考とし、当該試験においては 36 名のクロスオーバー試験にて有意差を得ていた。本試験において有意差を得るためには 22 名以上、36 名以下の被験者が必要と想定されたため本症例数を設定した。

16 倫理

本研究は、最新のヘルシンキ宣言の精神に基づき患者の人権および福祉を守り、文部科学省・厚生労働省「人を対象とする医学系研究に関する倫理指針」等のガイドラインを遵守して実施する。

16- 1 倫理審査

研究担当医師は、研究の実施に当たって、研究代表者所属施設の倫理審査委員会の意見を聴き、研究機関の長の許可を受ける。

16- 2 個人情報等の保護

本研究実施に関わる者は、被験者のプライバシーおよび個人情報の保護に十分配慮し、適正に取り扱う。登録された被験者情報は本研究用の登録番号を付与することで匿名化を行う。報告書には氏名、番号など個人を特定できる情報は記載しない。研究の結果を公表する際は、被験者を特定する情報を含まない。研究で得られた被験者のデータは、本研究の目的以外には使用しない。本研究の目的以外で使用する場合は、必要に応じ別途対象者から同意を取得する。

16-3 健康被害補償

本研究に起因して、被験者に何らかの健康被害が生じた場合には、研究実施施設は治療その他必要な措置を講じる。治療に係る費用は被験者が加入する健康保険により支払われる。

16-4 被験者の利益と不利益

本研究の結果は、食品成分の摂取により食後の熱産生・エネルギー消費の効率を改善させることで、エネルギーの摂取と消費のバランスを改善する方法が確立される可能性がある。エネルギー摂取と消費のバランスの改善は肥満や加齢に伴う様々な疾患の予防に重要であり、今後の医学の発展に繋がることが期待される。

本研究で使用される試験食は日常の食事で摂取する範囲内のものであり不利益を被ることは極めて少ないと考える。

16- 5 被験者の費用負担

本研究で実施する研究対象者の試験の費用は、順天堂大学・新潟大学・ブルボンの共同研究費から供出される。

最後まで協力いただいた被験者には協力費（測定参加 7,000 円/日×2 日＋試験食摂取負担費 6,000 円＝20,000 円のクオカードを支給する。

支払いに関しては受払簿、領収書にて適正に管理する。

17 品質管理・品質保証

17- 1 データの取り扱い

試験データは、順天堂大学において、研究の中止あるいは研究の終了報告日から１０年間、保管される。

17- 2 記録の保存

被験者の同意文書、被験者番号対応表等の関連文書、検査データ、倫理審査委員会の承認書など研究に関する情報は、研究実施施設にて研究の中止あるいは研究終了 10 年後 にはすべて廃棄いたします。に保管される。

17- 3 モニタリングおよび監査

【モニタリング実施について】

自主モニタリングを開始時、変更時、終了時および年 1 回以上実施する。

自主モニター：順天堂大学先進老化制御学講座　非常勤助教　古内　亮

【監査実施について】

監査は実施しない。

モニタリング実施にあたっては、「人を対象とする医学系研究に係るモニタリング・監査の実施に関する手順書」（臨床研究支援センター）に従って実施する。

17- 4 研究機関の長への報告

1) 研究者等は、研究に関連する情報の漏えい等、研究対象者等の人権を尊重する観点または研究の実施上の観点から重大な懸念が生じた場合には、速やかに研究機関の長に報告する。
2) 研究者等は、研究の実施の適正性若しくは研究結果の信頼を損なう事実若しくは情報または損なうおそれのある情報を得た場合には、速やかに研究機関の長に報告する。
3) 研究責任者は、研究の倫理的妥当性若しくは科学的合理性を損なう事実若しくは情報または損なうおそれのある情報であって研究の継続に影響を与えると考えられるものを得た場合(4) に該当する場合を除く)には、遅滞なく、研究機関の長に対して報告する。
4) 研究責任者は、研究計画書に定めるところにより、研究の進捗状況及び研

究の実施に伴う有害事象の発生状況を研究機関の長に報告する。

5) 研究責任者は、研究を終了(中止の場合を含む)したときは、研究機関の長に必要な事項について報告する。

18 研究資金および利益相反

18.1　研究資金

本研究は株式会社ブルボンが出資する順天堂大学・新潟大学・株式会社ブルボンの共同研究費を利用し実施される。また、本研究にはブルボンが出資する順天堂大学共同研究講座に所属する者がいる。

18.2　利益相反

本研究の研究代表者および分担者には、資金提供者である株式会社ブルボン所属者及びブルボンが出資する順天堂大学の共同研究講座に所属する者がいる。しかし、測定実施、データ解析にはブルボンとは直接的な利益相反関係にない新潟大学担当者が実施しブルボンが関与しないようにする。また、全てのデータ解析が終了するまでは盲検化を維持することで、研究結果が株式会社ブルボンに有利に歪められることがないようにする。

なお、各研究者は、所属学会および所属施設の利益相反マネジメントポリシーに従って適切に COI を管理し、研究成果を発表する学会や医学雑誌の求めに応じて開示することとする。

19 研究成果の公表および権利の帰属

19- 1 臨床試験登録

本研究は UMIN- CTR(http: //www.umin.ac.jp/ctr/index- j.htm) に登録し、情報公開する。臨床試験登録は、最初の被験者の登録までに研究責任施設または研究事務局が行う。

19-2 研究結果の公表、成果の帰属

研究代表者は、本研究終了後、遅滞なくその結果を公表する。研究成果を発表する者は、事前に研究代表者、主任研究者および研究班の審査・承認を得る。論文・学会発表等の著者は、International Committee of Medical Journal Editors の著者要件(authorship)に従い、 研究代表者と主任研究者が適切に決定する。

本研究で得られたすべてのデータは、研究組織に帰属する。

20 研究実施計画書の遵守と変更

20-1 研究実施計画書からの逸脱

研究担当医師は、被験者の緊急の危険を回避するなど、医療上やむをえない場合を除き、順天堂大学医学部医学系研究等倫理委員会の事前承認に基づく研究機関の長の承認を得る前に、研究実施計画書から逸脱または変更を行ってはならない。

21 研究の終了および中止・中断

21-1 研究の終了

研究の終了時には、研究担当医師は研究機関の長に文書により報告する。

21-2 研究の中止・中断

研究実施施設の判断や事由により研究を中止または中断した場合、研究担当医師は速やかに研究代表者に報告する。

【研究中止時の対応】

研究責任者または研究分担者は、次に挙げる理由で個々の研究対象者について研究継続が不可能と判断した場合には、当該研究対象者についての研究を中止する。その際は、必要に応じて中止の理由を被験者に説明する。また、中止後の研究対象者の治療については、研究対象者の不利益とならないよう、誠意を持って対応する。

【中止基準】

① 研究対象者から研究参加の辞退の申し出や同意の撤回があった場合
② 妊娠が判明した場合
③ 本研究全体が中止された場合
④ その他の理由により、研究担当者が研究の中止が適当と判断した場合

22.研究対象者等及びその関係者が研究に係る相談を行うことができる体制及び相談窓口

研究対象者等及びその関係者からの相談については、下記相談窓口にて対応す

る。

【相談窓口】

研究責任者　順天堂大学 循環器内科学講座　准教授　清水逸平
〒113-8421　東京都文京区本郷 2-1-1

新潟大学　新潟大学人文社会科学系（教育学部）天野達郎
〒950-2181　新潟県新潟市西区五十嵐 2 の町 8050 番地

株式会社ブルボン　前島大輔
〒950-2181　新潟県新潟市西区五十嵐 2 の町 8050 番地

23 参考文献

1) N Engl J Med 2009; 360:1500-1508
2) Int J Obes (Lond). 2021 Nov;45(11):2499-2505.
3) J Nutr Biochem. 2019 Feb;64:1-12
4) British Journal of Nutrition (2010), 103, 775–780
